# Mathematical Modeling and Analyses of Interspike-Intervals of Spontaneous Activity in Afferent Neurons of the Zebrafish Lateral Line

**DOI:** 10.1038/s41598-018-33064-z

**Published:** 2018-10-05

**Authors:** Sangmin Song, Ji Ah Lee, Ilya Kiselev, Varun Iyengar, Josef G. Trapani, Nessy Tania

**Affiliations:** 1 Department of Biology and Neuroscience Program, Amherst College, Amherst, MA 01002 USA; 2 Department of Mathematics and Statistics, Smith College, Northampton, MA 01063 USA

## Abstract

Without stimuli, hair cells spontaneously release neurotransmitter leading to spontaneous generation of action potentials (spikes) in innervating afferent neurons. We analyzed spontaneous spike patterns recorded from the lateral line of zebrafish and found that distributions of interspike intervals (ISIs) either have an exponential shape or an “L” shape that is characterized by a sharp decay but wide tail. ISI data were fitted to renewal-process models that accounted for the neuron refractory periods and hair-cell synaptic release. Modeling the timing of synaptic release using a mixture of two exponential distributions yielded the best fit for our ISI data. Additionally, lateral line ISIs displayed positive serial correlation and appeared to exhibit switching between faster and slower modes of spike generation. This pattern contrasts with previous findings from the auditory system where ISIs tended to have negative serial correlation due to synaptic depletion. We propose that afferent neuron innervation with multiple and heterogenous hair-cells synapses, each influenced by changes in calcium domains, can serve as a mechanism for the random switching behavior. Overall, our analyses provide evidence of how physiological similarities and differences between synapses and innervation patterns in the auditory, vestibular, and lateral line systems can lead to variations in spontaneous activity.

## Introduction

Temporal patterns of activity vary between the auditory, vestibular, and lateral line systems due to differences in synaptic physiology and connectivity between hair cells and afferent neurons. In the absence of stimuli, hair cells spontaneously release neurotransmitter that generates spontaneous action potentials (spikes) in innervating afferent neurons^1–4^. Properties of spontaneous spiking patterns are typically quantified through analysis of interspike-intervals (ISIs)^5^. Through mathematical modeling, recent studies have suggested that spontaneous ISI patterns from the auditory system are governed in part by depletion of synaptic vesicles at the readily releasable pool of vesicles at specialized ribbon synapses^4,6,7^. However, the extent to which synaptic depletion may also impact spontaneous activity within the vestibular or lateral line systems is less characterized. The synaptic arrangement of afferent neurons of inner hair cells in the auditory system is in contrast to vestibular and lateral line neurons that make multiple synaptic contacts onto multiple hair cells^8–10^. This difference in connectivity raises the question of whether synaptic arrangement plays a role in the diversity of temporal patterns of spontaneous spikes seen across systems. Here, we examined spontaneous activity from the lateral line of larval zebrafish to determine whether the spike patterns of this system could be described by the depletion model of the auditory system.

During mechanotransduction, activation of voltage-gated calcium channels (VGCCs) leads to an influx of calcium, synaptic vesicle fusion at ribbon synapses, and release of glutamate from the hair cell into the synaptic cleft. Upon glutamate binding to postsynaptic receptors, the afferent neuron is depolarized, reaches threshold at the spike generator, and an action potential is initiated. Spontaneous spikes in afferent neurons are also generated by neurotransmitter release from hair cells, presumably from the random opening of VGCCs^3,11–13^ with the rate and pattern of spikes determined by both presynaptic and postsynaptic processes. The postsynaptic mechanisms are especially evident in the vestibular system where regular and irregular classes of afferent neurons display tonic and phasic (respectively) spike patterns based on differences in their synaptic connectivity, ion channel expression, and intrinsic excitability^8,14–17^.

In the auditory system where synaptic innervation is one-to-one between a single hair cell and a single post-synaptic neuron, spontaneous spiking is proposed to be strongly affected by synaptic depletion that results in negative serial correlations of ISIs^4,6,7^. That is, the time required to replenish vesicles at a single ribbon synapse limits the frequency of synaptic release and therefore, the timing of spontaneous spikes in the innervating afferent neuron. These single synaptic connections result in a temporal pattern of spontaneous spiking that is strongly dependent on the depletion state of an individual hair cell. In contrast to the auditory system, the multiple synaptic contacts of vestibular and lateral line neurons led us to hypothesize that spike patterns would be less constrained by synaptic depletion since other innervating and non-depleted synapses could still drive spiking in the innervating afferent neuron. To test this hypothesis, we analyzed patterns of spontaneous spiking recorded from afferent neurons in the zebrafish lateral line by quantifying the underlying characteristics of ISI distributions and the correlation between consecutive ISIs.

Using renewal processes, we considered three different distributions for synaptic release time and compared the resulting ISI distributions to our electrophysiological data. These distributions for synaptic release time qualitatively represent distinct mechanisms, namely (i) random generation of synaptic release, (ii) release under the possibility of synaptic pool depletion, and (iii) independent and multiple sources of synaptic release. We also explored and extended the depletion computational model of synaptic release and depletion (referred to as the “depletion-replenishment” model from here on) previously used to describe spontaneous spiking in the auditory system^4^. We found that synaptic pool depletion cannot explain ISI patterns observed in the zebrafish lateral line. Specifically, by coupling several synaptic inputs within the depletion-replenishment model, we found that simulated data were stripped of negative serial correlations. However, increasing the number of innervated synapses per afferent neuron did not account for other differences in ISI properties between the auditory and lateral line systems. Thus, ISI patterns seen in lateral line neurons are distinct from those observed in the auditory system and may be more similar to the activity of irregular neurons in the vestibular system. Altogether, variations in synaptic physiology between the different sensory systems can lead to distinct patterns in spontaneous activity that may serve for unique information transfer dictated by the different systems^18,19^.

## Results

### Comparison of ISI data to a Poisson Process

**Figure 1:**
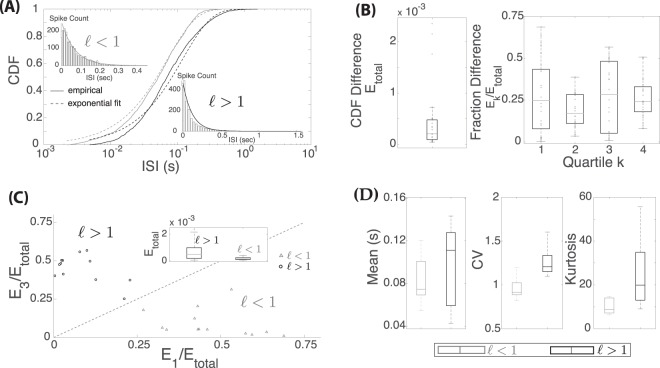
Comparison of ISI Distribution to Exponential Distributions. (**A**) Distributions of two ISI data sets in comparison to fitted exponential distributions. (**B**) Differences between empirical and best-fit exponential CDFs, *E*_*total*_ and the fractional difference by quartiles *E*_*k*_/*E*_*total*_. (**C**) Clustering of data by CDF differences in the first quartile *E*_1_ versus the third quartile *E*_3_. (**D**) Box plot of mean, CV and kurtosis of ISI data sets with $\ell$ < 1 (in gray) and $\ell$ > 1 (black).

In order to analyze the temporal patterns of spontaneous spiking in afferent neurons of the lateral line, we began by fitting each ISI distribution to an exponential distribution. We saw two general shapes of ISI distributions: one with a more exponential-shape and another with an “L”-shape. The exponential-shaped ISI distributions only deviated from the exponential-estimator for short ISI values (due to the refractory period of the afferent neuron). In contrast, the L-shaped distributions had a faster rate of decay and a heavier tail when compared to its exponential distribution estimator. To observe these two characteristic behaviors, we plotted the cumulative distribution function (CDF) and corresponding histogram of two different data sets (Fig. 1A).

For the exponential-shaped ISI distribution, the largest deviation between the empirical and exponential CDFs is seen in the first quartile, but this difference tapers off so that the two CDFs match closely in the third and fourth quartiles. However, for the L-shaped distribution, large deviations between the two CDFs are seen not only for short ISIs, but also for ISIs in the upper second quartile and third quartile. We then plotted the total deviation between the empirical CDF and the fitted exponential CDF, *E*_*total*_ and the fraction of the total difference for each ISI quartile *E*_*k*_/*E*_*total*_ (as defined in Eq. (6)) across all datasets (Fig. 1B). For a given data set, either *E*_1_ or *E*_3_ values could be highest, therefore we used the ratio of *E*_3_ to *E*_1_ ($\ell ={E}_{3}/{E}_{1}$, see Methods) to characterize the shapes of the two ISI distributions (Fig. 1C). Distributions that were more exponential in shape had $\ell$ < 1 (13 data sets out of 26) and L-shaped distributions had $\ell$ > 1 (13 datasets out of 26). In Fig. 1D, we also compared the values of the mean, coefficient of variation (CV), and kurtosis for the two distribution shapes. For comparison, exponentially distributed random variables have CV = 1 and kurtosis = 9. L-shaped distributions tended to have a wider spread in ISI values as reflected in their higher CV and kurtosis values.

### Comparison of ISI data to renewal processes

Given that spontaneous spiking is driven by neurotransmitter release from hair cells, we hypothesized that the contrast between the two observed shapes of ISI distributions is caused by differences in synaptic release patterns of innervated hair cells. Thus, we next turned to renewal process models, commonly used for modeling neuronal spiking^6,7,20–23^, that explicitly separate the refractory period of the afferent neuron and the waiting time for synaptic release (excitation timing). For the model considered here, the renewal interval (equivalent to each ISI) consists of two parts: the refractory period (consisting of a constant absolute-refractory period followed by an exponentially-distributed relative-refractory period), and the excitation (synaptic release) time. The resulting theoretical ISI distribution *f*_*ISI*_(*t*) can be found by the convolution integral (8). We considered three distributions (cases (i–iii) below) for the excitation wait time in order to qualitatively test different underlying mechanisms that govern synaptic release (see Methods and Supplementary Materials).

**Case (i) - Exponential** *f*_*E*_(*t*)**:**

1 $${f}_{E}(t)={\lambda }_{E}\,\exp \,(\,-\,{\lambda }_{E}\,t)$$

**Figure 2:**
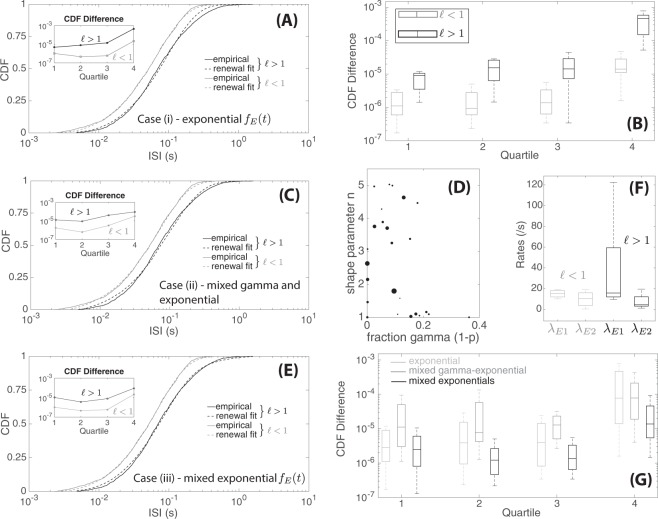
Data-fitting to renewal equations with three different excitation time distributions *f*_*E*_(*t*). (**A**) Empirical CDFs of two ISI data sets (same data sets as in Fig. 1A) in comparison to the theoretical CDF of *f*_*ISI*_(*t*) (Eq. (8)) with exponentially-distributed excitation time *f*_*E*_(*t*) (case (i)). (**B**) Differences between empirical and best-fit CDFs (as defined in Eq. (6)) for case (i) by quartiles for datasets ($\ell$ < 1 in gray and $\ell$ > 1 in black). (**C**) Empirical CDFs of two ISI data sets in comparison to theoretical CDF with gamma-exponential mixture excitation time (case (ii), same data sets as in (A)). (**D**) Best-fit values for 1 − *p*, the fraction events generated by the gamma distribution, against *n*, the gamma shape parameter. Dot size corresponds proportionally to the total difference between the empirical CDF and model CDF (larger dots correspond to larger differences). (**E**) Empirical CDFs of two ISI data sets in comparison to renewal CDF with two exponentials mixture excitation time (case (iii), same data sets as in (**A**)). (**F**) Box plots of best-fit rate parameters values *λ*_*E*1_ and *λ*_*E*2_ for datasets ($\ell$ < 1 in gray and $\ell$ > 1 in black). (**G**) Comparisons of total difference in empirical and theoretical CDFs (as defined in Eq. (6)) for for the three different models considered (case (i–iii)).

For the first case, we assumed that synaptic release events are randomly generated at a constant rate *λ*_*E*_. In Fig. 2A, we show two representative fits between the model and empirical CDFs. All ISI data with $\ell$ < 1 (exponential-shaped) showed good fits with the resulting *f*_*ISI*_ distribution, while fits for $\ell$ > 1 data (L-shaped) were less consistent. We then found the total square-difference between the model CDF and the empirical CDF at each quartile (Fig. 2B). Datasets with $\ell$ < 1 had an overall lower difference, by approximately one order of magnitude, when compared to those with $\ell$ > 1. However for both shapes, the highest error was observed in the fourth quartile, indicating that there were a higher number of longer ISIs in the data than predicted by this renewal process model. To further understand the cause for the higher fraction of longer ISIs and the different shapes of ISI distributions, we expanded our starting assumption and considered two different mixture distributions for excitation wait time. For case (ii), we qualitatively tested the possibility of synaptic depletion, and for case (iii), we considered the possibility of distinct sources of synaptic release.

**Case (ii) - Gamma-Exponential Mixture** *f*_*E*_(*t*):

2 $${f}_{E}(t)=p\,[{\lambda }_{E}\,\exp \,(\,-\,{\lambda }_{E}\,t)]+(1-p)\,[\frac{{\lambda }_{E}^{n}}{{\rm{\Gamma }}(n)}\,{t}^{n-1}\,\exp \,(\,-\,{\lambda }_{E}\,t)]$$

The gamma-exponential mixture model was previously used to represent the effects of synaptic depletion^6,7^. Here, a synaptic release event is generated either by an exponential distribution with rate *λ*_*E*_ with probability *p* (as in case (i)), or a gamma distribution with rate *λ*_*E*_ and shape parameter *n* with probability 1 − *p*. Note that a gamma distribution with an integer-valued shape parameter *n* describes a waiting time for *n* events to occur (where each event occurs according to an exponential distribution). If *n* = 1, *f*_*E*_ reduces fully back to case (i), but if *n* > 1, the resulting distribution would be shifted in the direction of longer ISIs. We specifically considered this mixture model as it was previously used to describe the distribution of ISIs in the auditory system^6,7^. There, the shape parameter value was fixed at *n* = 2 and the gamma distribution was used to mimic the effects of synaptic depletion in that it took two exponentially-distributed “events” (*n* = 2, failure of one event) to generate an excitation signal for some fractions of ISIs. Here, we allowed *n* to take on any value above one and analyzed the resulting fit.

Despite the addition of two free parameters, *n* and *p*, we were not able to obtain a better fit compared to case (i). As shown in Fig. 2C, the resulting fits between this model and the empirical CDFs were fairly similar to those in case (i). Moreover, the best-fit parameter values for the fraction of gamma-generated events, 1−*p*, was small (on average, $1-p \sim 0.1$); that is, most of the excitation times were generated by the exponential distribution and only a small fraction were due to the gamma distribution that represents the synaptic depletion effect (Fig. 2D). Thus, this mixture model gave very similar fits to those obtained in case (i), with only exponentially-distributed excitation times. Our data fitting results suggest that synaptic depletion, at least as modeled by the gamma-exponential mixture distribution for excitation time, could not fully explain neither the higher fraction of longer ISIs seen in our data nor the difference in ISI distribution shapes.

**Case (iii) - Two Exponentials Mixture** *f*_*E*_(*t*):

3 $${f}_{E}(t)=p\,[{\lambda }_{E1}\,\exp \,(\,-\,{\lambda }_{E1}t)]+(1-p)\,[{\lambda }_{E2}\,\exp \,(\,-\,{\lambda }_{E2}t)]$$

Given the synaptic arrangement of the lateral line where a single afferent neuron makes multiple synapses onto multiple hair cells, we next considered the possibility of distinct sources for synaptic release events. To accomplish this, we utilized a mixture distribution consisting of two exponential distributions of different rates *λ*_*E*1_ and *λ*_*E*2_ (with proportion *p* and 1 − *p* respectively) to describe the timing of the excitation signal; for convention, we take *λ*_*E*1_ > *λ*_*E*2_. Representative fits of this model to the empirical CDF are shown in Fig. 2E. Comparisons of resulting best-fit parameter values showed that for datasets with $\ell$ < 1, the values of *λ*_*E*1_ and *λ*_*E*2_ tended to be closer to each other (on average, *λ*_*E*1_ ≈ 2 × *λ*_*E*2_) (Fig. 2F). In contrast, when $\ell$ > 1, *λ*_*E*1_ tended to be much higher than *λ*_*E*2_ and the difference could be up to one order of magnitude (Fig. 2F). This large difference in rates suggests that L-shaped ISI distributions occur when one source of release generates synaptic events more quickly than others.

In order to compare the goodness of fit between the three models tested, we plotted differences between the model and empirical CDFs (Fig. 2G). Comparing the three cases, the difference between the model and empirical CDFs was lowest for the exponential-mixture model (case (iii)) across all quartiles (also see Supplementary Materials: total CDF differences for each dataset are listed in Tables S1–S3 and two-sample t-tests between each model were performed indicating that two exponential mixture model having the smallest CDF difference). In particular, the difference in the fourth quartile decreased by almost one order of magnitude, showing that this model better captured the fractions of longer ISIs seen in the data. To examine the tradeoff between additional parameter values and goodness of fit, we computed the Akaike Information Criterion and Bayesian Information Criterion (see Supplementary Materials for details of computation and Table S4 showing results) and found that both criteria gave similar conclusions: in most instances, case (iii) where hair cell synaptic releases were modeled with a mixture of two exponential distributions, was most consistent with the data but for a few exceptions where case (i) (exponentially distributed *f*_*E*_(*t*)) was deemed more consistent.

### Relationship between consecutive ISIs

**Figure 3:**
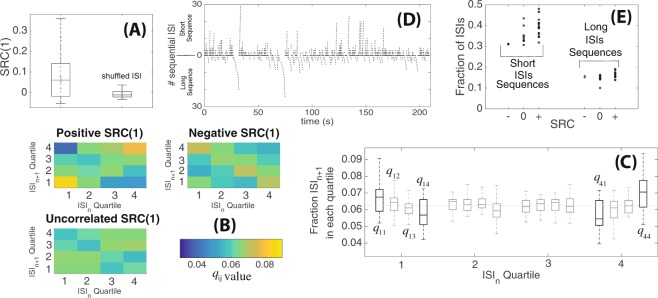
Dependencies between sequential ISIs. (**A**) Box plot of SRC for consecutive ISI pairs (*SRC*(1)) for original unsorted data sets and shuffled data set. (**B**) Visualizations of the renewal quartile matrix *q*_*ij*_ showing frequencies for an ISI at a given quartile (horizontal axis) to be followed by an ISI at another quartile (vertical axis). Three qualitatively different mappings can be seen for data sets with positive correlation, zero correlation and negative correlations. (**C**) Boxplot of entries of renewal quartile matrices across all data sets. Compared to an equal likelihood for any ISI to fall in any quartile (*q*_*ij*_ = 1/16 shown as dashed line), we saw overall increases in *q*_11_ (short followed by short) and *q*_44_ (long-long), while *q*_14_ (short-long) and *q*_41_ (long-short) were reduced. (**D**) Groupings of long and short ISIs that occur consecutively in sequence over time. (**E**) Fraction of ISIs that are found within long and short sequences for data sets with negative, zero (uncorrelated), and positive *SRC*(1) (each dot represent a dataset).

In our analyses thus far, we had not considered how consecutive ISIs may be dependent on each other. Consecutive ISIs in the auditory system were previously shown to be negatively correlated, so that shorter ISIs tended to be followed by longer ISIs and vice versa, which was predicted to result from synaptic pool depletion^4^. Thus, we next looked at the relationship between sequential ISIs in our lateral-line data. We first computed the serial correlation coefficient for two sequential ISIs, *SRC*(1) (see (9) with *n* = 1) and found that 15/26 datasets showed positive serial correlation (8 were uncorrelated and just 3 exhibited negative correlation; also see Figs S4–S6 in the Supplementary Materials for p-values). Generally, a positive *SRC*(1) means a long ISI, compared to the mean, tends to be followed by another long ISI and correspondingly, a short ISI tends to be followed by another short ISI. When the orderings between ISIs within each dataset are shuffled, *SRC*(1) goes to zero (Fig. 3A).

As SRCs are calculated by comparing to the mean of the entire spike train, we next refined our measurements by dividing the ISI distribution into quartiles and computing the recurrence quartile matrix *q*_*ij*_ (as defined in Methods). If there were no dependencies between sequential ISIs (*SRC*(1) ≈ 0), there would then be an equal likelihood for the next ISI to land in any of the quartiles regardless of the quartile of the previous ISI, so *q*_*ij*_ ≈ 1/16 = 0.0625. On the other hand, a higher value of *q*_14_ would indicate that an ISI in the first quartile (shortest 25% of the ISIs) tends to be followed by an ISI in the last quartile (longest 25%) and so on. In Fig. 3B, we visualized three typical recurrence quartile matrices: for positively correlated data, *q*_11_ and *q*_44_ tended to be higher, for negatively correlated data, *q*_14_ and *q*_41_ were higher instead, and for uncorrelated data the recurrence quartile matrix appeared more uniform (*q*_*ij*_ ≈ 1/16). When we analyzed the *q*_*ij*_ values across the entire data set (Fig. 3C), we found that *q*_11_, *q*_14_, *q*_41_, *q*_44_ tended to deviate most from 1/16. Higher *q*_11_ and lower *q*_14_ values indicated that a short ISI tended to be followed by another short ISI. Lower *q*_41_ and higher *q*_44_ values also showed a similar trend for long ISIs (i.e., consecutive long ISIs). These observations were consistent with the fact that we most often observed positive serial correlations in our lateral line data.

Looking beyond the correlation for two sequential ISI pairs, we next asked if short or long ISIs tended to be clustered together over time. We adapted a method for identifying bursting activity^24^ and computed how many short or long ISI pairs occurred sequentially as follows. First, let *μ*_*short*_ and *μ*_*long*_ denote the mean of all short ISIs (below the global mean) and all long ISIs, respectively. A finite *sequence* of short (or long) ISIs is said to occur if the average ISI within the sequence was shorter (or longer) than *μ*_*short*_ (or *μ*_*long*_, respectively). In Fig. 3D, we show a rastergram for spike times from a single recording but we grouped several spikes together (upward/downward trends in the plot) if their corresponding ISIs formed a sequence of long/short ISIs. We saw that there were indeed time periods when short and long ISIs were grouped together. Across our data sets, we found that approximately ~30% of ISIs occurred within sequences of short ISIs and ~15% in sequences of long ISIs.

The fraction of ISIs found in long or short ISI sequences were higher for data with positive *SRC*(1) than those with negative or uncorrelated *SRC*(1) (Fig. 3E). Thus, long (or short) ISIs were more likely to occur sequentially in data with positive *SRC*(1), though sequences of long and short ISIs were nonetheless seen across all our datasets. We also examined whether ISI data with L-shaped distributions ($\ell$ > 1) exhibited a different serial relationship than those with exponential shape ($\ell$ < 1). Interestingly, we found that the L-shaped data had a higher positive *SRC*(1) than those with $\ell$ < 1 and the tendency for a short ISI to be followed by another short ISI (or long/long) was higher L-shaped data (Fig. S1 in Supplementary Materials). We next also looked at Fano factor and *SRC*(*n*) with longer lag *n* to further quantify long term dependencies between ISIs.

### Fitting Using Simple Switching Model

Our analyses of sequential ISIs suggested that ISIs tend to be grouped over time such that shorter (or longer) ISIs follow one another in sequence. Moreover, data-fitting to renewal processes showed that the spontaneous spike patterns in the lateral line are best fitted with a model of synaptic release that consists of a mixture of two exponential distributions. We hypothesized that spontaneous spike generation shows a switching behavior between two different modes, one where spikes are generated more frequently and another less frequent mode. In the case of data with L-shaped distributions ($\ell$ > 1), the timescales (or rates) for spike generation between the two modes were quite different; thus we observed a more distinct sequencing of longer ISIs for some time periods and shorter ISIs in others. The effects of switching would be less prominent when $\ell$ < 1 (ISI data with more exponential shapes) since timescales for each mode were more similar. Thus, we would observe a narrower range of ISI values and “long” vs “short” ISI sequences became less distinguishable compared to the $\ell$ > 1 (L-shaped) case.

Consider the following two-state system,

4 $${S}_{slow}\underset{{k}_{fs}}{\overset{{k}_{sf}}{\rightleftharpoons }}{S}_{fast}.$$

During the slow state, *S*_*slow*_, ISIs are generated with exponentially-distributed synaptic release times with mean *τ*_*slow*_. During the fast state, *S*_*fast*_, the synaptic release occurs more frequently according to an exponential distribution with mean *τ*_*fast*_ (*τ*_*fast*_ < *τ*_*slow*_). The transition from the slow to fast state occurs at rate *k*_*sf*_ and from fast to slow at *k*_*fs*_. The proportion of time spent in *S*_*fast*_ is *p*_*fast*_ = *k*_*sf *_/(*k*_*fs*_ + *k*_*sf*_). Following each spike, the afferent neuron undergoes a refractory period (modeled as above, with a constant absolute refractory period followed by an exponentially-distributed relative refractory period). Neglecting the relationship between sequential ISIs, the distribution of ISIs in this model follows the previous renewal process with mixed-exponential synaptic release time (case (iii)) and has the following corresponding parameter values, *λ*_*E*1_ = 1/*τ*_*fast*_, *λ*_*E*2_ = 1/*τ*_*slow*_, and *p* = *p*_*fast*_).

**Figure 4:**
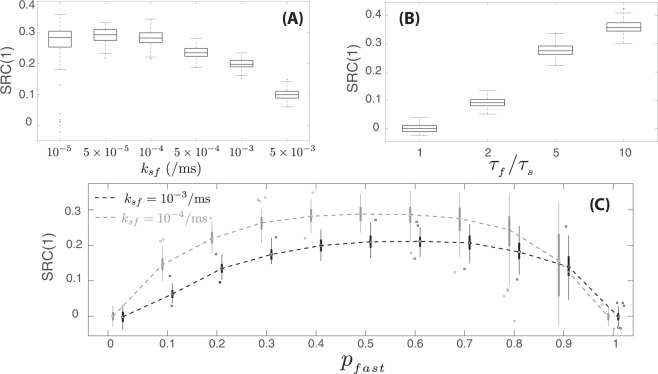
Dependencies of serial correlations for the two-state switching model on parameter values. (**A**) Box plots of *SRC*(1) values measuring correlations between consecutive ISIs for different values of switching rate *k*_*sf*_ (*k*_*fs*_ is varied so that *p*_*fast*_ is constant at 0.6, *k*_*fs*_ = *k*_*sf*_ (1−*p*_*fast*_)/*p*_*fast*_. All other parameter values were fixed: *p*_*fast*_ = 0.6, *τ*_*fast*_ = 40 ms, *τ*_*slow*_ = 200 ms, *t*_*abs*_ = 2 ms, and *t*_*rel*_ = 2 ms). (**B**) Box plots of *SRC*(1) values for different ratios of *τ*_*fast*_/*τ*_*slow*_ (all other parameter values were fixed: *k*_*sf*_ = 10^−4^/ms, *p*_*fast*_ = 0.6, *τ*_*fast*_ = 40 ms, *t*_*abs*_ = 2 ms, and *t*_*rel*_ = 2 ms). (**C**) Box plots of *SRC*(1) values for different values of *p*_*fast*_ using two different values of switching rates *k*_*sf*_ = 10^−3^/ms in black and *k*_*sf*_ = 10^−4^/ms in gray (all other parameter values were fixed: *τ*_*fast*_ = 40 ms, *τ*_*slow*_ = 200 ms, *t*_*abs*_ = 2 ms, and *t*_*rel*_ = 2 ms). For each box plot in this figure, 100 trials were performed with each trial consisting of 5000 ISIs.

We numerically simulated this switching model in order to explore how *SRC*(1) values were altered by varying parameter values. Our findings are summarized in Fig. 4. Increasing the rate of switching between states reduces *SRC*(1) values (Fig. 4A). If switching rates *k*_*sf*_ and *k*_*fs*_ are larger so that switching occurs more quickly (e.g., on the time scale of the refractory period or very few ISIs), the resulting *SRC*(1) would be closer to zero and sequential ISIs are uncorrelated. However, a positive *SRC*(1) becomes more likely with slower switching (time scale of multiple ISIs). More concretely, the longer the duration of time spent in state *S*_*slow*_ before a switching event occurs, the more likely that “longer” ISIs are generated sequentially since the synaptic excitation has an exponential wait-time with mean *τ*_*slow*_ under state *S*_*slow*_. Similarly, with a longer duration spent in *S*_*fast*_ (smaller rate *k*_*fs*_), sequential “short” ISIs become more likely. In addition to having switching rates that are not too large, a positive *SRC*(1) value also requires *τ*_*fast*_ to be significantly different than *τ*_*slow*_ (by convention *τ*_*fast*_ < *τ*_*slow*_) as shown in Fig. 4B. When *τ*_*slow*_ ≈ *τ*_*fast*_, switching between the two states would give ISIs that are of similar length yielding *SRC*(1) ≈ 0. Finally, we found the effects of changing the proportion *p*_*fast*_ with two different values of switching rates produced faster switching and smaller *SRC*(1) values (Fig. 4C). Sequential ISIs are also not correlated when *p*_*fast*_ is either zero or one, since no switching between the two states occurs (either *k*_*sf*_ or *k*_*fs*_ is zero). For 0 < *p*_*fast*_ < 1, *SRC*(1) is most positive at *p*_*fast*_ = 0.5 as there are equal proportions of time spent in *S*_*fast*_ and *S*_*slow*_. As *p*_*fast*_ was increased from 0 to 1, we also saw that *SRC*(1) values across trials became more varied (higher variance). Increasing *p*_*fast*_ led to a higher variance because the standard deviation of ISIs themselves would be higher during *S*_*fast*_ compared to *S*_*slow*_ (the mean and standard deviation of the exponentially-distributed excitation times for states *S*_*slow*_ and *S*_*fast*_ are respectively *τ*_*slow*_ < *τ*_*fast*_).

**Figure 5:**
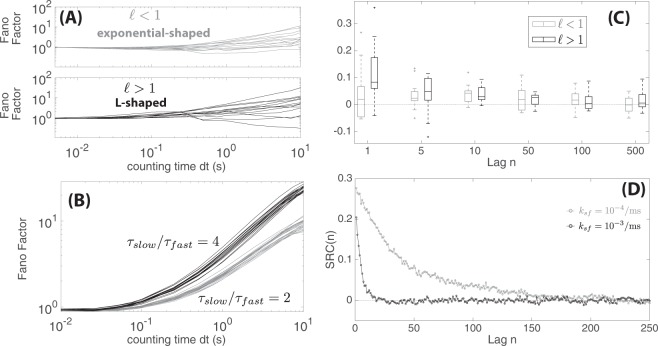
Long term dependencies between consecutive spikes in lateral-line data and the two-state switching model. (**A**) Fano factor as a function of counting time for the lateral line data (each curve corresponded to a dataset). (**B**) Fano factor obtained from simulating the two-state switching model using two different synaptic release time ratios: *top: τ*_*fast*_ = 40 ms, and *τ*_*slow*_ = 80/ms and *bottom: τ*_*fast*_ = 40 ms, and *τ*_*slow*_ = 160/ms. (All other parameter values were fixed: *k*_*sf*_ = 10^−4^/ms, *p*_*fast*_ = 0.4, *k*_*fs*_ = *k*_*sf*_ (1 − *p*_*fast*_)/*p*_*fast*_, *t*_*abs*_ = 2 ms, and *t*_*rel*_ = 2 ms). (**C**) *SRC*(*n*) at different lag time *n* (see definition in Eqn 9) for the lateral line data. (**D**) *SRC*(*n*) at different lag time *n* obtained from simulating the two-state switching model using two different values of switching rates *k*_*sf*_ = 10^−3^/ms in black and *k*_*sf*_ = 10^−4^/ms in gray (all other parameter values were fixed: *τ*_*fast*_ = 40 ms, *p*_*fast*_ = 0.4, *τ*_*slow*_ = 200 ms, *t*_*abs*_ = 2 ms, and *t*_*rel*_ = 2 ms). Each dot corresponded to the mean *SRC*(*n*) value from simulating 100 trials with each trial consisting of 2000 ISIs).

In order to further test out similarities between the two-state switching model and the lateral line data, we also analyzed dependencies between consecutive ISIs over longer time-periods as shown in Fig. 5. We first looked at the Fano factor, defined as the ratio of the variance to the mean number of spikes within a counting period *dt*. For a homogeneous Poisson process with constant rate (stationary process), the Fano factor will be one for any counting time. However, for non-stationary processes governed by rates that change over time (e.g. due to random drift in spiking rate or bursting), the Fano factor often increases as the counting time increases^4,25,26^, i.e., when a longer counting time is considered, non-stationary processes are less regular than a stationary Poisson process (higher variance compared to mean). As shown in Fig. 5A, the Fano factor computed for each lateral-line dataset also increases with counting time *dt* and we observed steeper increases for L-shaped datasets. For most of our data, the Fano factor exhibited a power law relationship (*F* ∝ *dt*^*n*^) for counting time *dt* < 1 s before starting to saturate similar to those seen in^18^; interestingly however, we also observed non power law behavior for some L-shaped data with positive SRC (see Fig. S2 in Supplementary Materials). A power-law like increase in the Fano factor could be attributed to a random drift in spiking rate^4,25^, but it could also be explained by our two-state switching model. In Fig. 5B, we showed Fano factor computations obtained from simulating the two-state switching model. We found that Fano-factor increases more steeply as a function of counting time when there were larger differences in synaptic release times between the two states (higher ratio of *τ*_*slow*_ to *τ*_*fast*_). We also previously showed in Fig. 2F, that indeed larger differences in time-scales were observed for L-shaped data (when fitted to the mixed exponential renewal model). Moreover, we also observed consistent behavior between the lateral-line data and the two-state switching model when the serial correlation *SRC*(*n*) was analyzed over longer lag *n* > 1. We found that ISIs separated by *n* intermediate spikes remain positively correlated even for lag *n* in the order of tens of ISIs (*SRC*(*n*) > 0 for *n* = 1, 5, 10, 50). These positive correlations were observed for both L-shaped and exponential data (either $\ell$ > 1 or $\ell$ < 1 respectively) as shown in Fig. 5C. Similarly, we found a more gradual decrease in *SRC*(*n*) when switching occurs more slowly (smaller *k*_*sf*_ and *k*_*fs*_ values) as shown in Fig. 5D.

The biological mechanism behind the switching behavior is currently unknown. Previously, Trapani and Nicolson^3^ hypothesized that both the mechanotransduction current, *I*_*MET*_ and the hyperpolarization-activated current, *I*_*h*_, contributed to maintaining the resting potential of hair cells within the lower-end of the activation range of voltage-gated calcium channels. This effect was proposed to result in the spontaneous influx of calcium and synaptic release from hair cells. The study also showed that ISI data could be described by a mixture of two exponential distributions with means *τ*_*fast*_ and *τ*_*slow*_. Blocking of either *I*_*MET*_ or *I*_*h*_ resulted in higher *τ*_*slow*_ values. However, the closings and openings of ion channels alone cannot fully explain the switching behavior observed here, since ion-channel activities occur at a faster time scale than multiple ISIs seen in our data. Potentially, cooperative openings of calcium channels can result in extended periods of higher calcium concentration and more frequent synaptic releases. Additionally, the innervation of a single afferent neuron by multiple hair cells may translate to a bulk switching behavior, especially if hair cells within a neuromast can be potentially coupled and/or are affected by synaptic depletion. We next tested the combined effects of multiple innervation and synaptic depletion by simulation with the depletion-replenishment model^4^.

### Simulations of the Depletion-Replenishment Model

**Figure 6:**
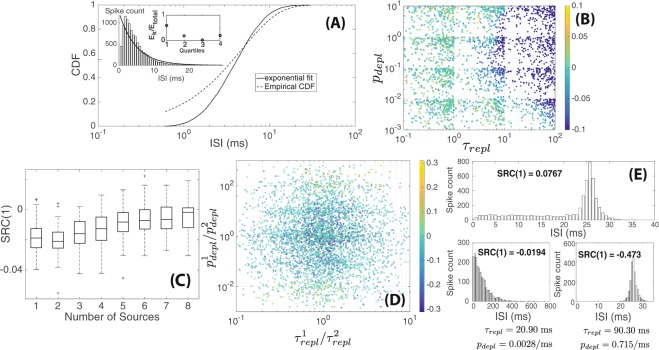
Simulation results from the depletion-replenishment model. (**A**) Comparison of ISI distribution generated from the model under synaptic depletion (*τ*_*repl*_ = 2.5 ms, *p*_*depl*_ = 0.08/ms, *n*_*max*_ = 4, 10,000 ISIs were generated) in comparison to the best-fit exponential distribution (analogous to Fig. 1A). Due to synaptic depletion, shorter ISI becomes less likely ($\ell =0.0352$). (**B**) Colormap of *SRC*(1) values obtained by random sampling of values for *τ*_*repl*_ and *p*_*depl*_ (*n*_*max*_ was fixed at 4). Each dot corresponded to a trial with 2000 ISIs generated; a total of 2000 dots/trials were generated. (**C**) The effects of varying the number of sources or hair cells on *SRC*(1) values. Each boxplot consisted of 100 trials where 2000 ISIs were generated per trial (*τ*_*repl*_ = 2.5 ms, *p*_*depl*_ = 0.08/ms and *n*_*max*_ = 4 for all sources). (**D**) Colormap of *SRC*(1) values obtained when parameters associated with the two different sources were allowed to vary from one another. Ratios of *p*_*depl*_ and *τ*_*repl*_ between the two sources are used as axis values. Each dot corresponded to a trial with 2000 ISIs generated; a total of 4440 dots/trials are shown. (**E**) *Top:* Shape of ISI distribution obtained with two sources with parameter values chosen to give rise to *SRC*(1) > 0. *Bottom:* Shape of ISI distributions if only the first or second source generated spikes in the absence of the other (${\tau }_{repl}^{1}\,=\,20.90$ ms, ${p}_{depl}^{1}\,=\,0.0028$/ms, ${\tau }_{repl}^{2}\,=\,90.30$ ms, ${p}_{depl}^{2}\,=\,0.715$/ms ${n}_{max}^{1}\,=\,{n}_{max}^{2}\,=\,4$; 2000 spikes were generated for each histogram).

To test whether some of the ISI characteristics seen in our lateral-line data could be explained by synaptic depletion effects, we simulated the depletion-replenishment model that was previously used to explain ISI behaviors in the auditory system^4^. We plotted a histogram of ISIs generated from this model with parameter values chosen to exhibit synaptic depletion (parameter values similar to original model^4^ with resulting *SRC*(1) = −0.027) (Fig. 6A). Here, shorter ISIs became less likely due to synaptic depletion, which resulted in the largest difference between the empirical CDF (simulation result) and the fitted exponential distribution occurring within the first quartile (resulting in $\ell$ ≪ 1). In the model, there are three parameters controlling the behavior of synapses namely *n*_*max*_, the maximum synaptic pool size, *p*_*depl*_, the probability rate of release for each synaptic pool and *τ*_*repl*_, the timescale of replenishment of each synaptic pool. Increasing *p*_*depl*_ and/or *n*_*max*_ leads to more frequent synaptic releases (until the pool is depleted) while increasing *τ*_*repl*_ leads to slower replenishment of the synaptic pool and thus less frequent releases. Given a specified maximum pool size *n*_*max*_, the effect of synaptic depletion would become more prominent when both *p*_*depl*_ and *τ*_*repl*_ are larger (i.e., more frequent release but slower replenishment). We then asked if there were parameter regimes that would lead to either L-shaped ISI distributions and/or ISI trains with positive *SRC*(1) as observed in our lateral-line data. We randomly generated 2000 pairs of *p*_*depl*_ and *τ*_*repl*_ over a wide range of values and computed the resulting values of *SRC*(1) and *l*. A colormap of the resulting *SRC*(1) values given a pair of values for *τ*_*repl*_ and *p*_*depl*_ showed that when *p*_*depl*_ and *τ*_*repl*_ are high, we observed negative *SRC*(1) due to synaptic depletion (Fig. 6B). Otherwise, we found *SRC*(1) values close to 0 (uncorrelated serial ISIs).

Within the parameter set sampled in Fig. 6B, we did not observe any L-shaped distributions ($\ell$ ≪ 1 under synaptic depletion or $\ell \approx 1$ otherwise). While the connection between an afferent neuron and hair cell is a one-to-one synapse in the auditory system, afferent neurons make multiple synapses within the lateral line. Allowing for more than one of these synapses to be functional at a given moment, we simulated the depletion-replenishment model with multiple “sources” (synapses) for synaptic release. When each source is likely to experience synaptic depletion (large *p*_*depl*_ and *τ*_*repl*_), increasing the number of sources led to *SRC*(1) values becoming less negative (Fig. 6C). Thus, multiple sources ultimately removed the effects of synaptic depletion experienced by each source and yielded an uncorrelated sequential SRC.

Finally, we asked if heterogeneities in sources (hair-cell synapses) could lead to behaviors that might explain ISI characteristics seen in our lateral line data, namely positive *SRC*(1) values. In prior simulations (Fig. 6C), we assumed that all sources had the same parameter values. Here, we considered the case of only two synapses with varying parameter values: ${p}_{depl}^{1}$, ${p}_{depl}^{2}$, ${\tau }_{repl}^{1}$ and ${\tau }_{repl}^{2}$ (assume *n*_*max*_ = 4 for both sources). A colormap of the resulting *SRC*(1) values from sampling over a wide range of parameter values reveals that a positive *SRC*(1) value can be generated when the values of *p*_*depl*_ widely varies from one source to the other (e.g. ${p}_{depl}^{1}\gg {p}_{depl}^{2}$ or vice versa) (Fig. 6D). From the 4400 trials generated (4400 random combinations of parameters), 160 of them (or 3.6% of cases) have strongly positive *SRC*(1) > 0.05.

In Fig. 6E (top panel), we plotted a histogram of ISIs obtained using a set of parameter values that yielded a positive *SRC*(1). The resulting shape of the ISI distribution was different from what we observed in the lateral line, but it also could no longer be approximated by an exponential distribution. This shape resulted from the combination of the ISI distributions from each individual source. One source (bottom left panel of Fig. 6E) had a faster replenishment (larger *τ*_*repl*_) but less frequent release (smaller *p*_*depl*_) and its ISIs were more exponentially distributed (this source generated the shorter ISIs seen in the simulation with two sources). The second source (bottom right panel of Fig. 6E) had a slower replenishment but much faster release rate; interestingly, the resulting histogram appeared more normally distributed as fast release but slow replenishment resulted in less frequent generation of both shorter and longer ISIs. Our numerical simulations suggest that while heterogeneities in hair-cell synapses cannot fully explain all the behavior observed in the lateral-line data, they can yield ISI sequences with positive *SRC*(1) (albeit over a narrow range of parameter values in this computational model) and non-exponential ISI distribution-shapes.

## Discussion

By comparing the parameters needed to describe distributions of ISIs recorded from afferent neurons in the lateral line of zebrafish with those previously published for the auditory system, we demonstrated that the auditory depletion model does not account for the spike patterns of the lateral line system. Spontaneous activity from afferent neurons of the lateral line is uniquely described by two rates of spiking, one fast and one slow, that together produce either exponentially-shaped or L-shaped distributions observed in our data. We quantified the L-shaped tendency of an ISI distribution by comparing the empirical cumulative distribution to the corresponding best-fit exponential distribution. We observed that L-shaped ISI distributions show the largest deviation for longer ISIs (fourth quartiles), while ISI distributions that are more exponential in shape display the largest deviation within the first quartile (i.e., short ISIs are less likely due to refractory periods of the afferent neuron). ISIs within the lateral line system also displayed positive serial correlations such that shorter ISIs tend to be followed by similarly short ISIs and correspondingly, long ISIs are followed by long ISIs. We also found that groups of short (or long) ISIs tended to occur in sequence, which may indicate a switching between faster or slower regimes of spike generation. Using numerical simulations, we showed that a simple switching model can yield ISI distributions and serial relationships similar to those observed in our lateral line data. This switching behavior is similar to bursting though there are several key differences. First, we do not observe any multi-modal ISI distributions which are commonly observed in bursting spike trains^27,28^. Moreover, a bursting spike train model was previously shown to have alternating positive-negative serial correlation, *SRC*(*n*), as *n* is increased^28^. In contrast, our switching model exhibited a positive *SRC*(*n*) that gradually decreased to zero with increasing lags (number of intermediate spikes).

Universally drifting spike rates on a longer timescale have been shown to produce a positive serial correlation in spontaneous activity of the auditory system^4,26^. While we cannot fully distinguished the effect of random drifts in our lateral line data, we found that the two-state switching model was able to explain several key characteristics of the ISI lateral-line data e.g. distribution shapes and serial correlation dependencies. It was possible that during our recordings, small movements in the medium may have resulted in different periods of hair-cell activation. However when mechanotransduction is abolished by either pharmacological block of MET channels or mutation, patterns of short and long ISIs were still present^3^. Alternatively, a possible biological mechanism by which two spiking rates may arise include fluctuations in the opening of VGCCs in hair cells^4^. Recent studies have suggested that release of neurotransmitter at the hair cell is governed by a calcium micro-domain, in which a collective input of a group of calcium channels influences a certain population of vesicles at the ribbon synapse^29–31^ (but see discussions on nano-domains^32,33)^. If lateral-line hair cells are controlled by a calcium micro-domain, strong fluctuations in calcium concentration would lead to an overall change in spike rate that could be described on average, by switching between two spiking rates.

Across sensory systems that utilize hair cells for transduction, there are three main types of synaptic contacts, with each imparting differences in the temporal patterns of spontaneous spiking. In the auditory system, a single afferent neuron makes a single bouton synapse with just one hair cell with differences in synapses and afferent neurons providing for different information transfer^13,34,35^. In primarily central regions of the vestibular system, Type I hair cells are innervated by irregular neurons via calyx synapses^14,36^, whereas in peripheral regions, Type II hair cells make bouton synapses with regular neurons^36,37^. Finally, dimorphic neurons, which are typically irregular, innervate both Type I and Type II hair cells^38^. For the lateral line system of fish and amphibians, clusters of hair cells (neuromasts) are innervated by ~2–4 afferent neurons with each neuron forming multiple bouton synapses with multiple hair cells of the same activation polarity^17,39–41^. In addition to their multiple synaptic arrangement, the extent to which the different synapses are functional versus silent may also vary and play a role in spontaneous activity patterns^17^. Ultimately, each form of synaptic arrangement will allow for sensitivity, specificity and selectivity, as well as synaptic redundancy, which may be more critical in some organs than others.

Spontaneous spike trains from afferent neurons innervating inner hair cells show negative serial correlations, with pairs of consecutive ISIs tending to follow short/long or long/short patterns. This result is hypothesized to be due to the effects of synaptic depletion outweighing other mechanisms by which a positive correlation might occur^4,6,7^. Here, we adapted the depletion-replenishment model^4^ to allow for each afferent neuron making multiple synaptic contacts, which is the typical arrangement for the lateral line and vestibular systems as mentioned above. Assuming that each synapse behaves similarly and independently from another, the effects of depletion become negligible when more active contacts are coupled to the afferent neuron. This innervation pattern leads to the possibility that other effects such as fluctuations in calcium concentration may become more significant. Additionally, our numerical simulations of the depletion model with multiple hair cell contacts suggest that heterogeneities in hair cell function (e.g. ion channel activity, calcium and synaptic ribbon regulation, etc.) can generate ISI distributions with shapes that deviate from the more classical exponential shape predicted by a purely Poisson process such as vesicle release at a single, non-depleting synapse. Our numerical simulations also showed that in the case where one synapse has a faster neurotransmitter release rate (while still being affected by synaptic depletion) than another, ISIs with positive serial correlations can be generated.

Our data analysis and computational simulations show that the patterns of spontaneous spiking within the lateral line system cannot be explained by the effects of synaptic depletion. Other factors inherent to a synapse arrangement that is not one-to-one like the auditory system, such as heterogeneities within the innervated hair cells may play crucial regulatory roles. Consequently, we propose that further investigation of the mechanism of spike generation, especially the effects of calcium domains, are needed to fully characterize the mechanisms governing the temporal patterns of spontaneous activity in the lateral line and vestibular systems. Developments of computational models that combine details of ion channel gating, synaptic release mechanism and synaptic arrangements^4,42–44^ may shed light on how each different factor can shape patterns of spontaneous spiking as well as evoked responses. Moreover, a comparative and detailed quantification of spontaneous activity in these systems can ultimately be used to highlight their physiological and mechanistic differences as compared to the auditory and other sensory systems, potentially highlighting the unique physiological role that spontaneous activity plays in each system.

## Methods

### Data Collection

Zebrafish experiments performed at Amherst College were approved by the Animal Care and Use Committee (IAUC) at Amherst College under assurance number 3925-1 with the Office of Laboratory Animal Welfare. Experiments performed at the Oregon Health and Science University were conducted according to the policies of the Institutional Animal Care and Use Committee of Oregon Health and Science University. Spontaneous spike data consisted of 26 recordings taken from 5 day old (d5) wild-type (WT) zebrafish larvae: each recording consisted of 2000 spikes (85–257 seconds in duration). To obtain the lateral-line afferent neuron recordings, zebrafish larvae were anesthetized, mounted and then paralyzed using 125 *μ*M *α*-bungarotoxin. Larvae were then rinsed and kept in normal extracellular solution containing 130 mM NaCl, 10 mM HEPES, 2 mM KCl, 2 mM CaCl2, 1 mM MgCl2, pH 7.8. Borosilicate glass pipettes were used for extracellular current recordings (pulled using P-97, Sutter Instruments; resistance between 5 and 15 MΩ). Recordings from a single afferent neuron were taken using an EPC 9 or 10 amplifier using Patchmaster software (Heka Elektronik) in loose-patch configuration (resistance between 20 and 80 MΩ). For each neuron, the electrode was placed next to the stereociliary bundles of a single neuromast that was first identified as phase-locking to 20-Hz sinusoidal stimulation with a fluid jet. Further details of the experimental methods can be found in^3,45^.

### Comparison to a Poisson Process

The empirical CDF *F*_*data*_(*t*) is defined as a piecewise step function so that given an ISI data ${\{{T}_{n}\}}_{n=1}^{M}$, sorted from shortest to longest (with *M* being the total number of ISIs in the data), then

5 $${F}_{data}(t)=\{\begin{array}{ll}0 & {\rm{for}}\,\,0\le t < {T}_{1},\\ n/M & {\rm{for}}\,\,{T}_{n}\le t < {T}_{n+1},\,\,(n=1,2,\mathrm{...}),\\ 1 & {\rm{for}}\,\,t > {T}_{M}\mathrm{.}\end{array}$$

Data were first compared to a simple waiting-time model, a Poisson process with exponentially-distributed wait times, whereby an event/spike occurs at a constant probability rate *λ*. Using the maximum likelihood estimator *λ* = 1/mean ISI, we calculated *E*_*total*_, the total square difference between the cumulative distribution function (CDF, denoted as *F*(*t*, *λ*)) of the fitted exponential distribution, and and the empirical cumulative distribution function (Empirical CDF, *F*_*data*_(*t*)) of the ISI data. To further refine our measurements, we also computed the difference for each ISI quartiles, *E*_*k*_ (*k* = 1, 2, 3, 4). That is,

6 $${E}_{total}=({E}_{1}+{E}_{2}+{E}_{3}+{E}_{4}),\,{\rm{where}}\,\,{E}_{k}={\int }_{{T}_{k-1}}^{{T}_{k}}\,{(F(t,\lambda )-{F}_{data}(t))}^{2}dt.$$

*T*_*k*_ is the ISI value at the end of the *k*-th quartile (e.g. *F*_*data*_(*T*_1_) = 0.25). If deviations between the exponential distribution and the data are equally likely to occur in any quartile, then *E*_*k*_/*E*_*total*_ ≈ 0.25.

Our ISI data deviated from the Poisson process model in several ways. For one, short ISIs are less likely to be observed due to refractory periods in the afferent neuron. Thus, the largest contribution to the difference *E*_*total*_ may likely be found in the first quartile (shortest 25% ISI’s), so *E*_1_/*E*_*total*_ > 0.25 but the ratio of *E*_2_, *E*_3_, *E*_4_ to *E*_*total*_ would be less than 0.25. However, we also observed large deviations in the third quartile for several ISI distributions within our data set (*E*_3_/*E*_*total*_ > 0.25). Visually, these distributions tended to be more L-shaped with a faster rate of decay and a heavier tail when compared to the exponential distribution. The L-shaped tendency was determined by computing

7 $$\ell ={E}_{3}/{E}_{1}.$$

### Data Fitting to Renewal Processes

After we compared our ISI data to a Poisson process, we turned to renewal processes as previously used in^6,7,20^. Here, each ISI is taken as the sum of the refractory period of the afferent neuron and the waiting time for an excitatory event (from hair cells) to trigger an action potential. Similar to^6,7^, we assumed a distribution for refractory time, *f*_*R*_(*t*), that consisted of a constant absolute refractory period, *t*_*abs*_, and a stochastic relative refractory period, *t*_*rel*_ (exponentially distributed with rate *λ*_*R*_). After recovery from the refractory period, the next action potential is triggered upon the arrival of excitatory input (above threshold) from synaptic release from hair cells. We considered three different possibilities for *f*_*E*_(*t*), the distribution of synaptic release time *t*_*E*_: (i) an exponential distribution, (ii) a mixture of gamma and exponential distributions, and (iii) a mixture of two exponential distributions. By combining the distributions of refractory and excitation time, the probability density function for ISI, *t* = *t*_*abs*_ + *t*_*rel*_ + *t*_*E*_, can be written by conditioning over possible total refractory time *t*_*R*_,

8 $${f}_{ISI}(t)={\int }_{{t}_{abs}}^{t}\,{f}_{R}({t}_{R})\,{f}_{E}(t-{t}_{R})d{t}_{R}.$$

For each model (case(i–iii)), we obtained parameter values by fitting the empirical CDF from data to the CDF of the renewal process model via least-square optimization and the difference between the two CDFs was recorded. The Akaike Information Criterion (AIC) and Bayesian Information Criterion (BIC) were also computed to measure the tradeoff between the number of free parameters and goodness of fit^46–48^. Further details on the data-fitting process and results are given in the Supplementary Materials.

### Dependence between sequential ISIs

For a Poisson process, events/spikes occur independently. Assuming a renewal process, the next spike time would be dependent upon the previous spike time (refractory period depends on the previous spike time) but the waiting time (ISI) would still be independent of each other. Moving beyond Poisson or renewal processes assumptions, we asked if consecutive ISIs were dependent on each other. Given a sequence of ISIs over time, ${\{IS{I}_{k}\}}_{k\mathrm{=1}}^{N}$, we quantify the dependence of sequential ISIs by computing the serial correlation coefficient *SRC*(*n*), the correlation coefficient between ISIs separated by *n* intermediate spikes.

9 $$SRC(n)=\frac{ < (IS{I}_{k+n}-\overline{ISI})(IS{I}_{k}-\overline{ISI}) > }{{\rm{Var}}(ISI)},$$

where $\overline{ISI}$ and Var(*ISI*) are the mean and variance of ${\{IS{I}_{k}\}}_{k\mathrm{=1}}^{N}$ respectively, while <⋅> denotes averaging. The first-order correlation *SRC*(1) describe dependencies between two directly consecutive ISIs. Higher-order correlations *SRC*(2), *SRC*(3), ... have been found to tend to zero as the lag (distance between ISIs) *n* increases^4,49^. We thus primarily focused on looking at the correlation between sequential ISIs, i.e. *SRC*(1), though we also found significant correlations for higher order SRC as shown in the Results section. To determine the significance of each SRC, the corresponding p-value (Pearson correlation) was calculated, assuming a null-hypothesis that ISIs are uncorrelated to each other (*SRC*(*n*) = 0).

SRC values are obtained by comparing each ISI to a single number, namely the global mean, $\overline{ISI}$. When looking at sequential ISIs, we further refined *SRC*(1) measurement by ranking ISIs by quartile. This was done by mapping sequential ISIs by their respective quartiles. Specifically, we define the ‘recurrence quartile matrix,” a 4 × 4 matrix with entries *q*_*ij*_, the fraction of data where the previous ISI lies in quartile *i* and the next ISI lies in quartile *j* (*i*, *j* = 1, 2, 3, 4). If there is no dependency between consecutive ISIs, then one can expect that the next ISI will have an equal likelihood of landing in any of the quartile regardless of the quartile of the previous ISI, i.e. *q*_*ij*_ ≈ 1/16 = 0.0625.

To further quantify dependencies of sequential ISIs, we also computed the Fano factor which is defined at the ratio of the variance to the mean number of spikes over a counting time period *dt*. The computation of Fano factor for our data set was performed by taking a sliding window of length *dt* and tabulating the corresponding number of spikes. For a homogeneous Poisson process with constant rate, *F*(*dt*) = 1 for all counting time period *dt*.

### Depletion-Replenishment computational model

To compare our lateral line data to the auditory system, we simulated the depletion-replenishment model^4^ that was previously shown to capture both the ISI distribution shape and negative *SRC*(1). We briefly summarize the features of the model here.

As in the renewal-processes, the refractory period of the neuron is assumed to consist of a constant absolute refractory *t*_*D*_ followed by a relative refractory period that is exponentially distributed with mean *t*_*R*_. No spike can be generated during the refractory period. Excitatory synaptic release from a hair cell is generated from a readily releasable “pool” of size *n*(*t*), which varies between 0 and a maximum number, *n*_*max*_. Each unit can be released independently with a probability rate *p*_*depl*_. By taking into account the pool size *n*(*t*), the probability rate of having a synaptic release at time *t* is *p*_*depl*_ ⋅ *n*(*t*). When a release event occurs, the pool size is reduced by 1 from its previous size. Then, the synaptic pool is replenished to its maximum size, *n*_*max*_, at a rate 1/*τ*_*repl*_.

We extended this model by considering multiple synapses or sources that each connect to a single afferent neuron. This was done by considering multiple pool sizes, *n*^*k*^(*t*) (*k* = 1, 2, ..., total number of sources), each with a maximum size ${n}_{max}^{k}$, a replenishment timescale ${\tau }_{repl}^{k}$, and a release probability rate ${p}_{depl}^{k}$. Each pool *k* is assumed to be independent of each other and can trigger a spike in the neuron with a probability rate ${p}_{depl}^{k}\cdot {n}^{k}(t)$. This model was implemented using a Monte-Carlo simulation with a step-size Δ*t* = 0.001 ms. At each time point *t*_*i*_ = *i* ⋅ Δ*t*, the possibility of a synaptic release at each source (*k* = 1, 2, …) was determined by drawing a random number *r*, from the uniform distribution in [0, 1] and checking whether $r < {p}_{depl}^{k}\cdot {n}^{k}({t}_{i})\cdot {\rm{\Delta }}t$. If a release event occurred, then the pool size is reduced by 1 so *n*^*k*^(*t*_*i*_) = *n*^*k*^(*t*_*i*−1_) − 1. Otherwise, the pool size continues to replenish to its maximum size ${n}_{max}^{k}$ according to^4^,

10 $${n}^{k}({t}_{i})={n}^{k}({t}_{i-1})+({n}_{max}^{k}-{n}^{k}({t}_{i-1}))\,[1-\exp \,(-\frac{({t}_{i}-{t}_{rel}^{k})}{{\tau }_{repl}^{k}})],$$

where ${t}_{rel}^{k}$ is the last release time for pool *k*.

## Electronic supplementary material

Supplementary Materials
