# Supplementary material for: Mathematical Modeling and Analyses of Interspike-Intervals of Spontaneous Activity in Afferent Neurons of the Zebrafish Lateral Line
Source: Sci Rep. 2018 Oct 5;8:14851. doi: 10.1038/s41598-018-33064-z (PMC6173758; doi:10.1038/s41598-018-33064-z)
Supplement: Supplementary file 1 — Supplementary Materials [file 41598_2018_33064_MOESM1_ESM.pdf]

Supplementary Materials

Mathematical Modeling and Analyses of Interspike-Intervals of Spontaneous Activity in Afferent Neurons of the Zebrafish Lateral Line

Sangmin Song[1], Ji Ah Lee[2], Ilya Kiselev[1], Varun Iyengar[1], Josef Trapani[1], and Nessy Tania[2,*]

[1]Department of Biology, Amherst College, Amherst, MA 01002, USA
[2]Department of Mathematics and Statistics, Smith College, Northampton, MA 01063, USA
[*]ntania@smith.edu

Renewal Process Models

Model Formulation

We model spike (action potential) generation as a renewal process consisting of two steps. A spike is generated if the afferent neuron is recovered *and* a synaptic release from hair cells (excitatory input) has occurred. Following an action potential, a neuron cannot immediately spike again and first undergoes a refractory period. We assumed that the refractory period consists of a constant absolute-refractory period followed by a relative refractory period. The duration of the absolute-refractory period, t_{abs}, is assumed to be a fixed constant that corresponds to the minimal time period before the neuron can undergo another action potential. The relative-refractory period, t_{rel}, is a random variable generated according to an exponential distribution with rate λ_R. Thus, the probability density function for the total refractory period is given by

$$f_R(t) = \begin{cases} 0 & \text{for } t < t_{abs}, \\ \lambda_R \exp(-\lambda_R(t - t_{abs})) & \text{for } t \geq t_{abs}. \end{cases} \tag{S1}$$

Once the neuron is recovered, it will remain at rest until a synaptic release arrives. This time to excitation, t_E, follows a probability distribution function $f_E(t)$; we considered three different distributions that qualitatively describe distinct mechanisms as discussed in the main article.

1. Exponential distribution with rate λ_E,

$$f_E(t) = \lambda_E \exp(-\lambda_E t). \tag{S2}$$

2. Mixture of Gamma and exponential distributions,

$$f_E(t) = p\left(\lambda_E \exp(-\lambda_E t)\right) + (1-p)\left(\frac{\lambda_E}{\Gamma(n)} t^{n-1} \exp(-\lambda_E t)\right). \tag{S3}$$

   The first term represents a fraction p of the excitation times generated by an exponential distribution with rate λ_E, while the second term represents the remaining fraction $(1-p)$ generated by a gamma distribution with shape parameter n and rate λ_E.

3. Mixture of exponential distributions,

$$f_E(t) = p\left(\lambda_{E1} \exp(-\lambda_{E1} t)\right) + (1-p)\left(\lambda_{E2} \exp(-\lambda_{E2} t)\right). \tag{S4}$$

   Here, we assume two independent sources of excitation times both of which exponentially distributed; the first source has rate λ_{E1} and probability p, the second with rate λ_{E2} and probability $1-p$.

By combining the distributions of refractory and excitation time, the probability density function for ISI, $t = t_{abs} + t_{rel} + t_E$, can be written by conditioning over possible total refractory time t_R,

$$f_{ISI}(t) = \int_{T_{abs}}^{t} f_R(t_R) f_E(t - t_R)\, dt_R. \tag{S5}$$

Least-Square Data Fitting

Parameter fitting to different f_{ISI} distributions above was completed via least-square minimizations on the cumulative distribution functions (antiderivatives of $f_{ISI}(t)$ (S5)). Our fitting procedure was similar to that outlined in Heil, et al. (2007). The absolute refractory period t_{abs} is assumed to be a constant whose value is the smaller of either 0.9×shortest ISI or 2.5 ms. For fitting to different excitation time distributions $f_E(t)$, we first performed data fitting for the exponentially distributed f_E (case (i)). This data-fitting results were then used as initial guesses for the more complicated mixture models. More specifically, we used the following initial guesses to start the least square minimization (implemented using lsqnonlin in Matlab):

1. Exponential distribution for $f_E(t)$
   We used the same choice of initial guesses as in Heil, et al. (2007).

$$\lambda_E^0 = \frac{1}{\text{mean ISI } - t_{abs}}, \qquad \lambda_R^0 = \sqrt{\max\{0, 1/(\text{Var(ISI)} - 1/\lambda_E^0)}$$

   Note that the result obtained by solving the convolution integral for f_{ISI} does not distinguish between λ_E and λ_R, i.e. interchanging the rates used in $f_E(t)$ and $f_R(t)$ yields the same expression for $f_{ISI}(t)$. Following data-fitting, we took the larger of the rates to be λ_R ($\lambda_E < \lambda_R$) giving a range of total refractory time (absolute plus relative) of $\sim 2-12$ ms. The resulting best-fit refractory parameters λ_R and t_{abs} were then used for the two mixture models below to limit the degree of freedom in data-fitting.

2. Mixture of Gamma and exponential distributions
   For initial guesses, we used the values of λ_E, λ_R from fitting to the exponential $f_E(t)$, a random number between 1 and 4 for the shape parameter n, and a random number, uniformly distributed between 0 and 1 for p. We tested 100 sets of initial guess values and ran least square optimization. The final parameter set with the smallest sum of square difference was chosen as the best-fit parameter.

3. Mixture of exponential distributions
   Initial guesses for the parameters were obtained from the results from fitting to the exponential $f_E(t)$: initially, λ_{E1} was set to λ_E obtained from the exponential data-fitting, λ_{E2} was obtained by multiplying λ_E (from exponential data-fitting) by a random number from 0 to 10, and p was initially set to a random number between 0 and 1. We again tested 100 sets of initial guesses and ran least square optimization from each. The final parameter set with the smallest sum of square difference was chosen as the best-fit parameter.

Best fit parameter values for each models are listed in Tables 1-3.

Model Evaluations

Once parameter fitting was done, we compared the CDF difference (SSD columns in Tables 1-3) and found that the CDF difference is smallest, across all data sets, for the mixed exponential CDF (case (iii)). We further tested this by performing one-tailed two-sample t-tests with the following alternative-hypothesis (null hypothesis that SSD are the same):

- H_1 : SSD for exponential $<$ SSD for mixed Gamma-exponential - rejected, $p = 0.0547$,
- H_1 : SSD for exponential $<$ SSD for mixed exponentials - accepted, $p = 0.0017$,
- H_1 : SSD for mixed Gamma-exponential $<$ SSD for mixed exponentials - accepted, $p = 0.0029$.

Additionally, we also evaluated the difference between the three models using the Akaike Information Criterion (AIC) and the Bayesian Information Criterion (BIC). These criteria are used to measure the tradeoff between the number of parameters and the goodness of fit of the model to the data.

Given an ISI dataset $\{ISI_k\}_{k=1}^N$, sorted from shortest to longest and N denoting the number of ISIs in the dataset, we define the empirical CDF as a piecewise linear function such that

$$F_{data}(ISI_k) = \frac{k}{N}. \tag{S6}$$

Denoting the model CDF as $F(t)$, we evaluated values of $F(t)$ at each ISI_k. The goodness of fit is measured using the log-likelihood function,

$$LL = -N \cdot \ln(\sqrt{2\pi}) - N \ln(\sigma) - \sum_{k=1}^{N} \frac{(F(ISI_k) - F_{data}(ISI_k))^2}{2\sigma^2}, \tag{S7}$$

where σ^2 represents measurement uncertainty (here taken to be a constant value of 0.1 across all datasets). This likelihood function assumes that all errors are independent and identically distributed with a normal distribution. The AIC and BIC is respectively defined as,

$$AIC = -2 \cdot LL + 2 \cdot K_p, \qquad BIC = -2 \cdot LL + K_p \cdot \ln(N), \tag{S8}$$

where K_p is the number of parameters involved in the model. The same refractory period parameter values (namely λ_R and t_{abs}) were used for all three models compared here. Thus, we only counted the number of parameters used in $f_E(t)$ for K_p, so $K_p = 1$ for the exponentially distributed $f_E(t)$ (case (i)), $K_p = 3$ for the gamma-exponential mixture (case (ii)), and $K_p = 3$ for the mixture of two exponentials (case(iii)). We determined which model is most consistent with the data by looking AIC and BIC values. A lower value (more negative) of AIC or BIC indicates a better model. We found that both AIC and BIC gave similar conclusions (see Table 4). In most cases, model (iii) with mixed exponential $f_E(t)$ is most consistent with the data though there were a few cases where model (i) is deemed most consistent.

Tables of Data Fitting Results

Dataset #	t_{abs} (ms)	$1/\lambda_R$ (ms)	$1/\lambda_E$ (ms)	SSD
1	2.5000	1.0000	103.8023	14.9960
2	1.5300	2.0350	85.3971	11.5371
3	1.8900	5.1875	64.2797	12.7117
4	1.3500	0.1000	30.0571	67.0271
5	2.5000	0.1013	85.2152	110.3327
6	2.1600	1.0000	45.2899	66.4405
7	2.2500	0.7068	52.9073	14.4063
8	2.5000	3.9863	105.2665	36.9623
9	1.9800	2.0546	68.7616	9.2877
10	2.5000	4.8986	98.8533	19.8469
11	2.4376	1.0000	50.5663	41.7124
12	2.5000	4.3005	96.8992	33.6167
13	1.3997	2.3255	79.1264	18.6114
14	1.0870	11.6052	85.8959	12.7329
15	2.5000	10.3001	97.6181	9.8741
16	2.5000	3.1309	122.4140	23.3867
17	2.0079	8.1880	55.3747	16.6694
18	0.7790	7.1572	90.5223	9.0096
19	2.5000	3.4343	63.3072	27.1732
20	2.5000	1.9739	90.6865	17.2356
21	2.5000	3.8392	65.1848	5.9885
22	2.3788	1.0000	33.6383	69.3876
23	0.7300	1.0000	86.9112	46.4528
24	2.0859	5.7215	56.1924	6.3252
25	2.4239	1.4399	76.2428	7.0868
26	1.9113	0.1000	100.4803	68.0964

Table S1. Best-fit parameter values for case (i) with exponentially distributed $f_E(t)$. SSD denote the sum of square difference, $SSD = \sum_{k=1}^{N}(F(ISI_k) - F_{data}(ISI_k))^2$.

Dataset #	t_{abs} (ms)	$1/\lambda_R$ (ms)	$1/\lambda_E$ (ms)	n (shape)	p (fraction exp)	SSD
1	2.5000	1.0000	95.4563	5.5339	0.9637	22.6599
2	1.5300	2.0350	84.3668	3.0757	0.9975	20.6141
3	1.8900	5.1875	64.2839	1.5721	0.9679	30.8084
4	1.3500	0.1000	25.0884	2.7713	0.8725	66.0503
5	2.5000	0.1013	69.9545	6.7263	0.8818	65.3558
6	2.1600	1.0000	41.2201	3.1396	0.8999	47.0824
7	2.2500	0.7068	44.5355	1.7351	0.7849	11.3063
8	2.5000	3.9863	76.0861	4.8635	0.8812	65.4366
9	1.9800	2.0546	57.6170	2.6005	0.8586	36.6013
10	2.5000	4.8986	98.6680	5.0442	0.9915	334.8282
11	2.4376	1.0000	51.9561	1.4064	0.9997	30.5427
12	2.5000	4.3005	89.7908	5.1367	0.9525	29.2498
13	1.3997	2.3255	76.3475	5.1744	0.9796	14.2559
14	1.0870	11.6052	89.6941	1.3458	0.9972	44.6663
15	2.5000	10.3001	98.5804	1.1613	0.5770	248.1164
16	2.5000	3.1309	122.8094	2.7397	0.9601	32.5143
17	2.0079	8.1880	54.7375	1.1121	0.3103	48.9654
18	0.7790	7.1572	90.2446	1.3355	0.9748	12.8438
19	2.5000	3.4343	58.1598	2.5551	0.9258	31.5468
20	2.5000	1.9739	79.6686	3.6880	0.9411	15.9124
21	2.5000	3.8392	65.8979	2.9681	0.9971	32.3334
22	2.3788	1.0000	27.8033	6.3103	0.8901	22.3474
23	0.7300	1.0000	73.5998	3.0517	0.8820	40.4161
24	2.0859	5.7215	58.6992	1.0071	0.9970	46.7645
25	2.4239	1.4399	68.5965	3.5201	0.9386	18.0411
26	1.9113	0.1000	87.9430	4.0820	0.9324	49.8319

Table S2. Best-fit parameter values for case (ii) with mixed gamma-exponential $f_E(t)$. SSD denote the sum of square difference, $SSD = \sum_{k=1}^{N}(F(ISI_k) - F_{data}(ISI_k))^2$.

Dataset #	t_{abs} (ms)	$1/\lambda_R$ (ms)	$1/\lambda_{E1}$ (ms)	$1/\lambda_{E2}$ (ms)	p_1 fraction from source 1	SSD
1	2.5000	1.0000	86.5052	167.2800	0.7192	7.2974
2	1.5300	2.0350	21.2770	86.3707	0.0101	10.9590
3	1.8900	5.1875	63.4518	114.8000	0.9775	12.6090
4	1.3500	0.1000	16.6083	85.2152	0.6159	18.0682
5	2.5000	0.1013	36.1141	239.4464	0.5311	28.9722
6	2.1600	1.0000	16.8848	75.5116	0.3576	9.4812
7	2.2500	0.7068	52.9073	55.9003	1.0000	14.4063
8	2.5000	3.9863	86.0585	340.5995	0.8349	21.5973
9	1.9800	2.0546	68.7569	68.7616	0.0277	9.2877
10	2.5000	4.8986	98.8533	98.8533	0.1244	19.8469
11	2.4376	1.0000	8.1773	59.1891	0.1087	6.1058
12	2.5000	4.3005	81.5461	369.4809	0.8663	22.9696
13	1.3997	2.3255	74.2776	961.3536	0.9615	12.0821
14	1.0870	11.6052	82.0749	237.3042	0.9531	11.1667
15	2.5000	10.3001	95.0209	4016.8709	0.9851	9.1513
16	2.5000	3.1309	104.2992	295.7705	0.8355	14.0650
17	2.0079	8.1873	55.3741	55.3741	0.1045	16.6695
18	0.7790	7.1572	88.3080	446.1895	0.9811	7.6650
19	2.5000	3.4343	54.0979	157.5274	0.8419	18.7378
20	2.5000	1.9739	82.9394	315.8859	0.9233	10.9598
21	2.5000	3.8392	65.1806	65.3424	0.9826	5.9885
22	2.3788	1.0000	10.6168	51.1876	0.2919	13.0114
23	0.7300	1.0000	63.5526	217.9029	0.7291	25.6208
24	2.0859	5.7215	56.1703	57.0679	0.9745	6.3252
25	2.4239	1.4399	74.8167	1758.7056	0.9892	5.8784
26	1.9113	0.1000	50.3195	228.3939	0.5347	11.4158

Table S3. Best-fit parameter values for case (iii) with mixed exponentials $f_E(t)$. SSD denote the sum of square difference, $SSD = \sum_{k=1}^{N}(F(ISI_k) - F_{data}(ISI_k))^2$.

Dataset #	AIC				BIC			
	Model (i)	Model (ii)	Model (iii)	AIC decision	Model (i)	Model (ii)	Model (iii)	BIC decision
1	-5995.5	-6090.2	-6226.4	(iii)	-5974.7	-6073.4	-6209.6	(iii)
2	-6143.4	-5797.7	-6178.1	(iii)	-6122.6	-5780.8	-6161.3	(iii)
3	-6055.6	-5704	-6065.7	(iii)	-6034.8	-5687.2	-6048.9	(iii)
4	-4750.9	-4793.3	-6014.4	(iii)	-4730.1	-4776.4	-5997.6	(iii)
5	-4143.8	-5115.8	-6119.2	(iii)	-4123	-5099	-6102.4	(iii)
6	-4801.7	-5282.3	-6095.1	(iii)	-4780.9	-5265.5	-6078.3	(iii)
7	-6144.8	-6035.1	-6140.8	(i)	-6124	-6018.3	-6124	(i)
8	-5661.4	-5775.6	-6186.9	(iii)	-5640.6	-5758.8	-6170.1	(iii)
9	-6157.1	-5818.7	-6153.1	(i)	-6136.3	-5801.9	-6136.3	(i)
10	-6070.1	-2136.2	-6066.1	(i)	-6049.3	-2119.4	-6049.3	(i) or (iii)
11	-5316.8	-5595.2	-6192.5	(iii)	-5296	-5578.4	-6175.7	(iii)
12	-5692.1	-6021.7	-6140.4	(iii)	-5671.3	-6004.9	-6123.6	(iii)
13	-5921.5	-6014.6	-6158.9	(iii)	-5900.7	-5997.8	-6142.1	(iii)
14	-6105.8	-6013.2	-6187.1	(iii)	-6085	-5996.4	-6170.3	(iii)
15	-6068.3	-2939.9	-6150.4	(iii)	-6047.5	-2923.1	-6133.6	(iii)
16	-5873.5	-5842.3	-6199	(iii)	-5852.7	-5825.5	-6182.2	(iii)
17	-6005.4	-5995.4	-6001.4	(i)	-5984.6	-5978.6	-5984.6	(i) or (iii)
18	-6062.4	-5867	-6124.1	(iii)	-6041.7	-5850.2	-6107.3	(iii)
19	-5844.5	-5770.8	-6159.8	(iii)	-5823.7	-5754	-6143	(iii)
20	-5969.2	-6156.6	-6195.5	(iii)	-5948.4	-6139.8	-6178.7	(iii)
21	-6182.1	-6161.4	-6178.1	(i)	-6161.3	-6144.6	-6161.3	(i) or (iii)
22	-4772.5	-6048.6	-6070.9	(iii)	-4751.7	-6031.8	-6054.1	(iii)
23	-5469.5	-5680.9	-6147	(iii)	-5448.7	-5664.1	-6130.2	(iii)
24	-6180.2	-5488.3	-6176.2	(i)	-6159.4	-5471.5	-6159.4	(i) or (iii)
25	-6133.7	-5928.6	-6196.8	(iii)	-6112.9	-5911.8	-6180	(iii)
26	-4802.5	-5179.6	-6185	(iii)	-4781.7	-5162.8	-6168.2	(iii)

Table S4. AIC and BIC for the three models.

Additional Figures

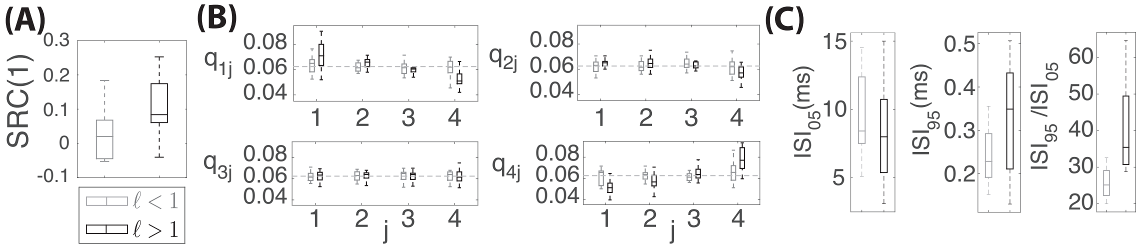

Figure S1. **Increase in serial dependencies for ISI data with L-shaped distribution ($\ell > 1$).** (A) Box plot of SRCs for data sets with $\ell < 1$ and $\ell > 1$. (B) Comparisons of entries of renewal quartile matrix q_{ij}. (C) Measurements of short ISI values (5th-percentile, ISI_{05}), long ISI values (95th-percentile, ISI_{95}), and the ratio between the two.

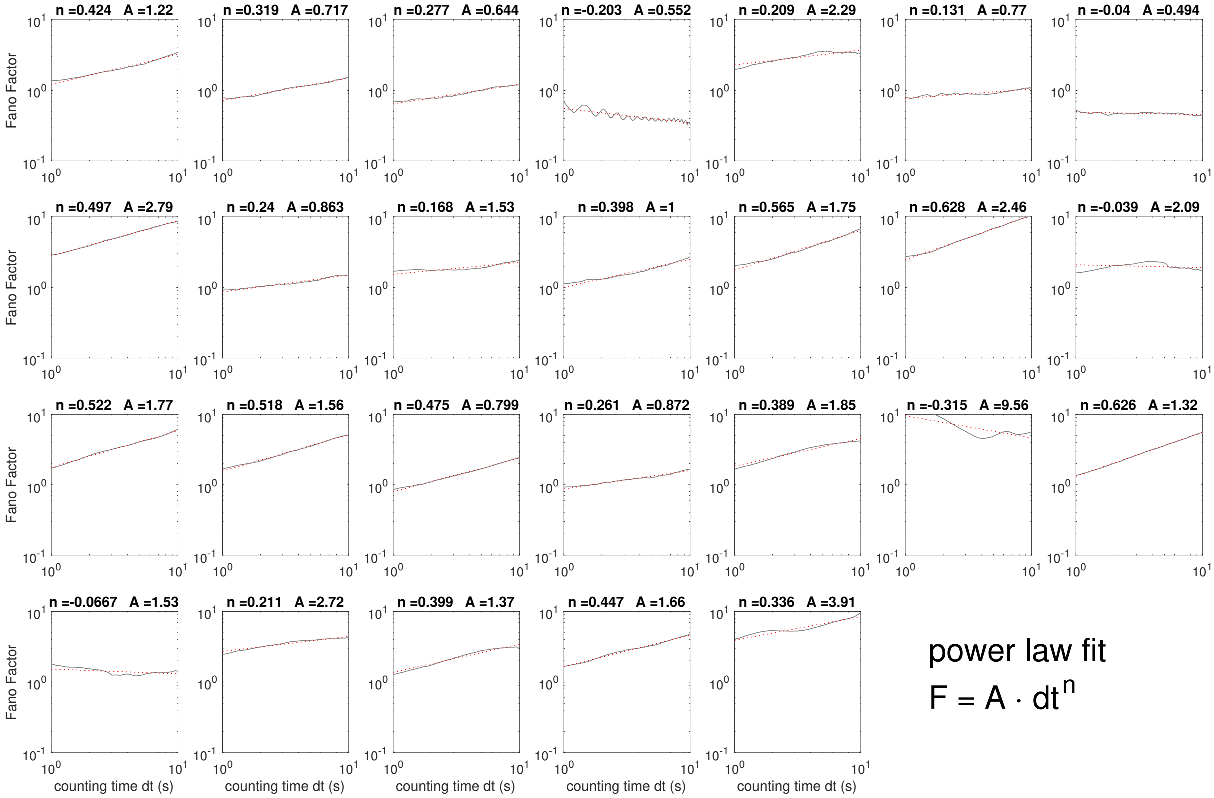

Figure S2. **Fano factor dependencies over counting time dt fitted to a power law relationship $F = A \cdot dt^n$.**

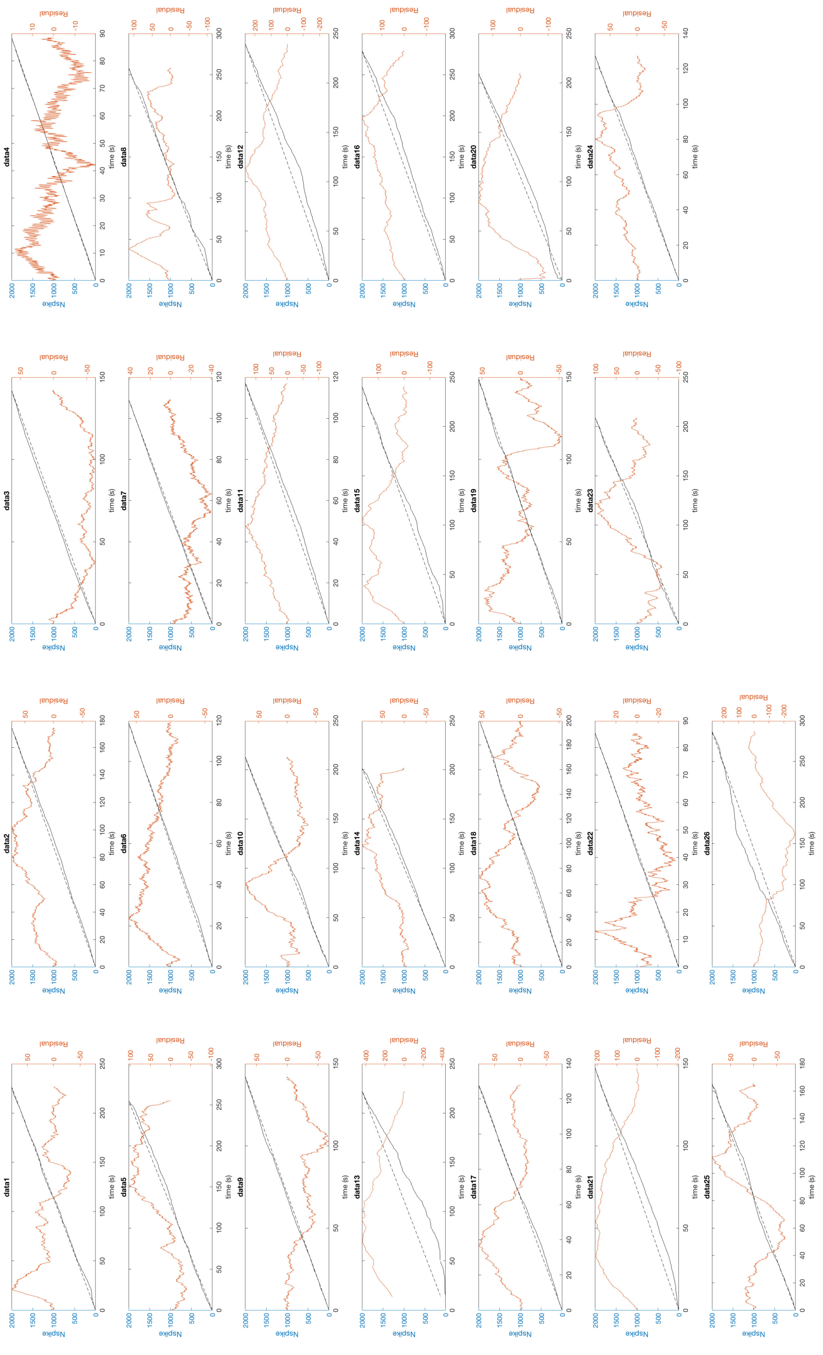

Figure S3. Comparison of spike count data (solid black line) to a Poisson process (dashed black line) with a linear spike count over time ($N_{spike} = \lambda t$ where λ is the spike rate ($\lambda = 1/\text{mean ISI}$ for fitting here). The difference between the model and the Poisson approximation is shown as the residual curve in red (see[?] Figure 4B for similar analysis).

Histograms of Data Sets

SRC values listed on histograms on Figs. S4-S6 corresponds to lag $n = 1$, ($SRC(1)$).

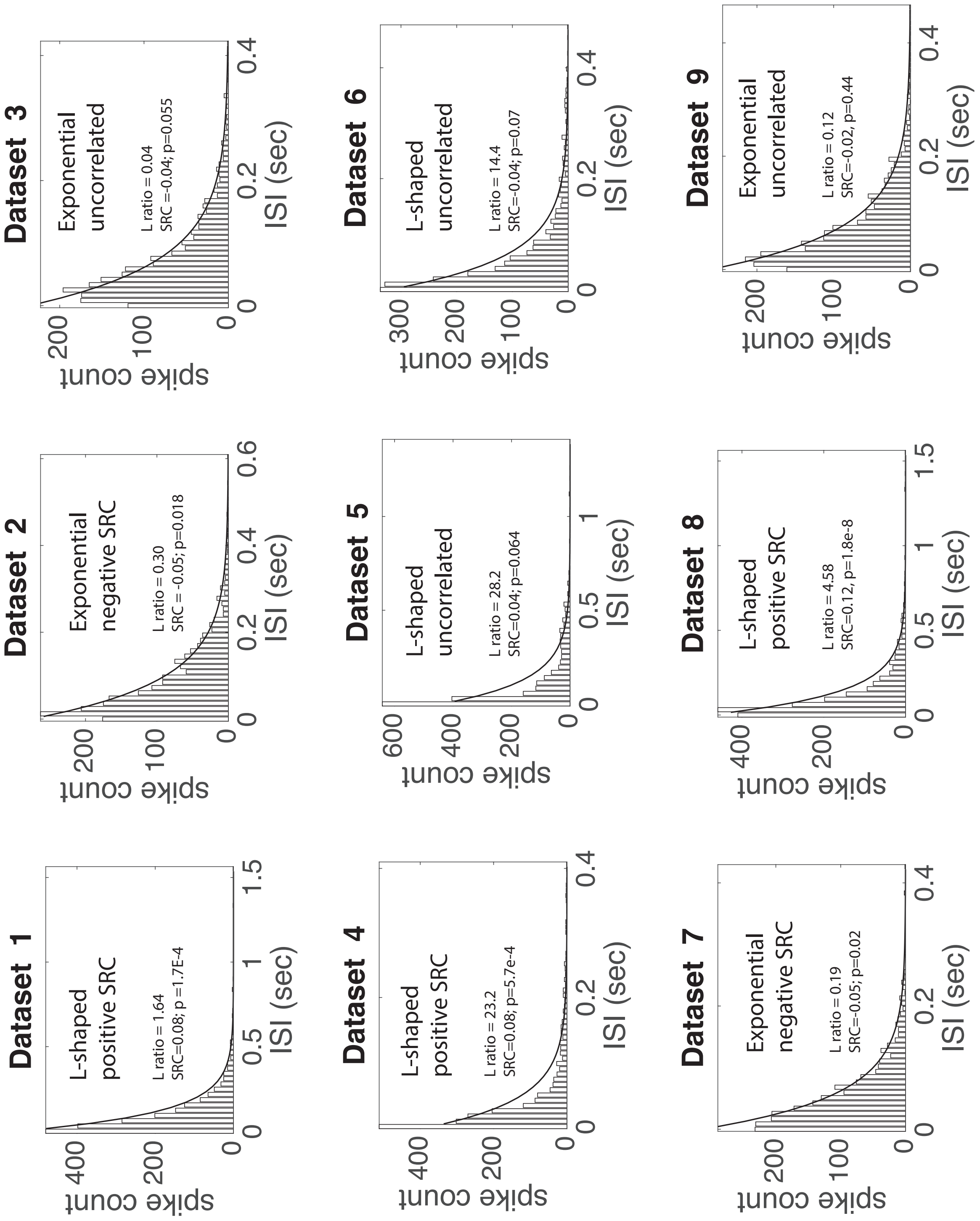

Figure S4. Histogram of Data Sets 1-9

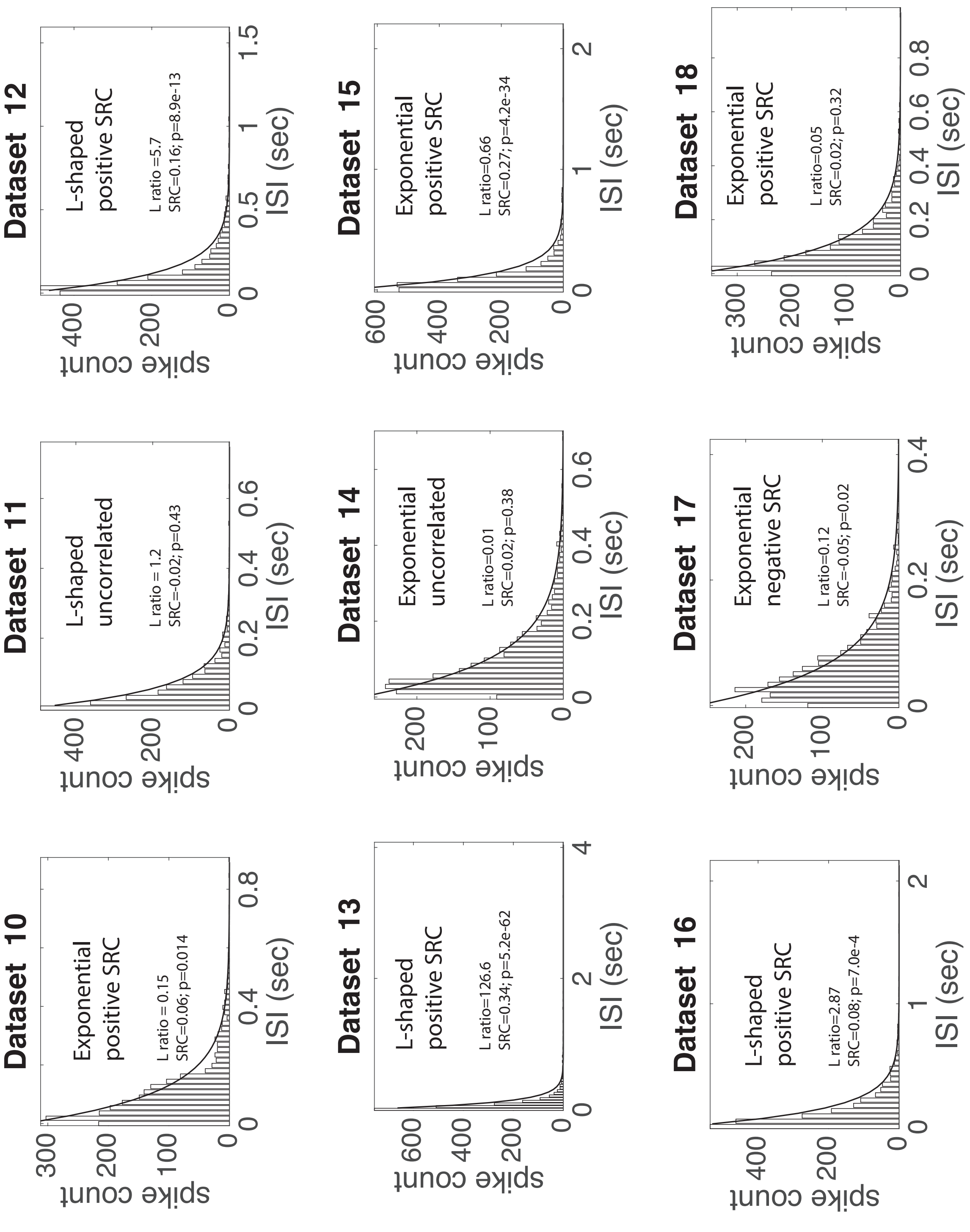

Figure S5. Histogram of Data Sets 10-18

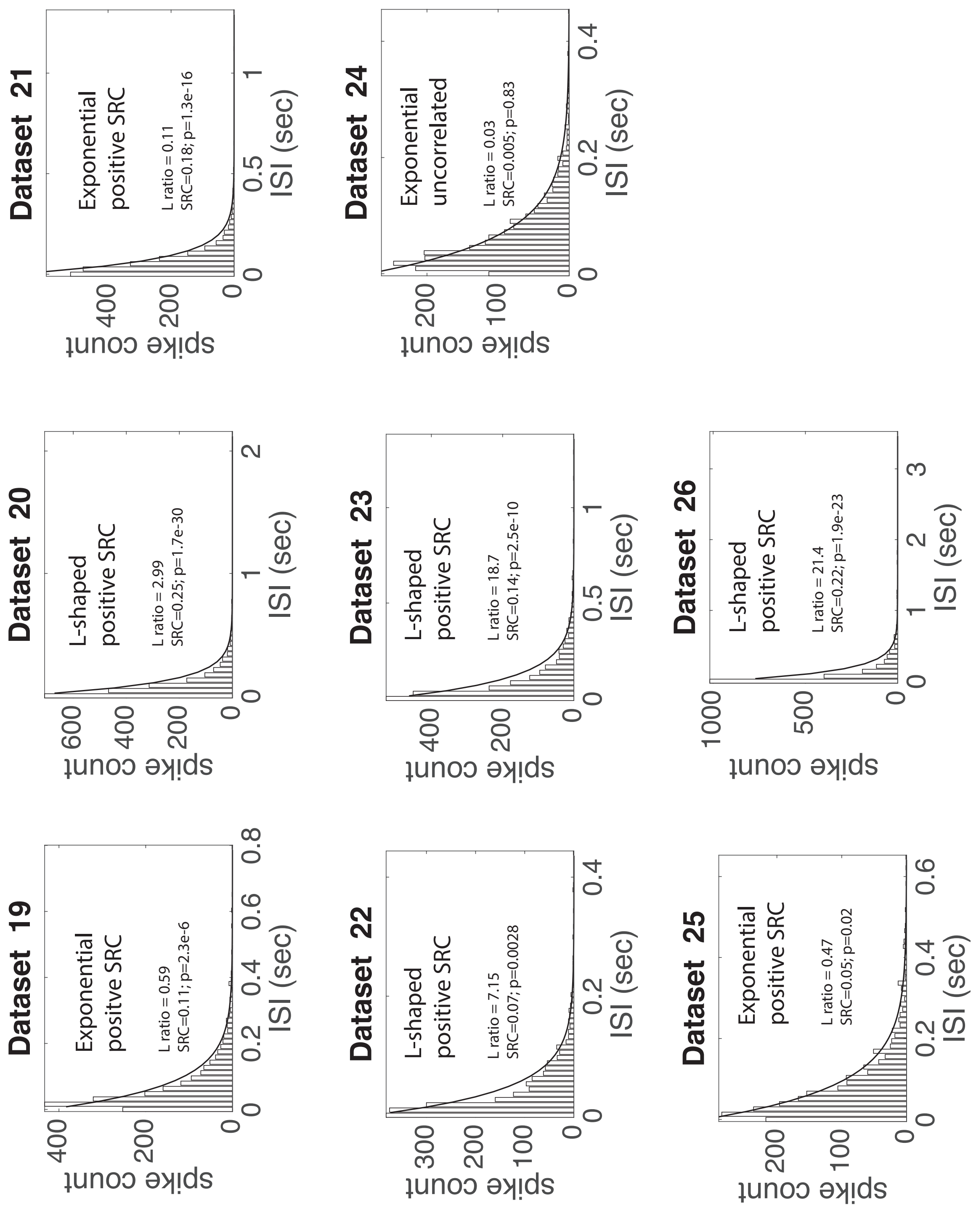

Figure S6. Histogram of Data Sets 19-26
